# Unlocking Immune Tolerance for Alzheimer's Therapy: A Treg‐Inducing Vaccine Alleviates Disease Burden in a Mouse Model

**DOI:** 10.1002/alz70861_108673

**Published:** 2025-12-23

**Authors:** Thura Tun Oo, Monserrat Hernandez‐Reyes

**Affiliations:** ^1^ Department of Radiology, University of Wisconsin‐Madison School of Medicine and Public Health, Madison, WI USA; ^2^ National Autonomous University of Mexico, Mexico City, EM Mexico

## Abstract

**Background:**

Alzheimer’s disease (AD) involves persistent neuroinflammation that accelerates neurodegeneration. Regulatory T cells (Tregs) are key suppressors of immune activation, yet their function is often impaired in AD. Restoring Tregs activity aims to re‐establish immune homeostasis and presents a potential therapeutic target for AD (1). We investigated a Treg‐inducing vaccine to therapeutically reinstate tolerance and reduce AD pathology in vivo.

**Method:**

In this study, we evaluated the anti‐AD efficacy of two novel tolerogenic adjuvant compositions developed in our laboratory. We analyzed brain T cell responses, neuroinflammation, amyloid‐beta (Aβ) deposition, and cognitive function in immunized mice. To facilitate comprehensive T cell analysis, we generated and characterized a novel APP/FoxP3‐eGFP/Ki67‐tagRFP (“AFK”) mouse model. The genotype of AFK mice was confirmed using polymerase chain reaction. Following an initial period on a normal diet (ND), the mice were randomly divided into three groups (*n* =6 per group) and treated as follows: 1) phosphate‐buffered saline as a mock control; 2) adjuvant composition A; or 3) adjuvant composition B. Post‐treatment, the mice remained on ND for an additional four weeks, after which behavioral assessments were conducted. Then, the mice were euthanized, and their brains were analyzed for relevant pathological parameters.

**Result:**

Our findings demonstrate that both tolerogenic adjuvant compositions A and B mitigated cognitive decline and reduced amyloid‐beta (Aβ) deposition in the brains of treated mice. Notably, composition B exhibited superior efficacy, enhancing cognitive function by approximately 40% and reducing Aβ deposition by approximately 30%. Analysis of neuroinflammatory markers revealed significant modulation of key parameters, including brain tumor necrosis factor‐alpha and interleukin‐6, across the treatment groups. Furthermore, flow cytometric analysis of brain tissue from treated mice revealed that composition B induced a substantial increase (approximately 7‐fold) in the ratio of regulatory T cells (Tregs) to effector T cells (Teffs).

**Conclusion:**

Collectively, these results indicate that tolerogenic adjuvant composition B possesses greater therapeutic potential against AD in this preclinical model. Future investigations are warranted to further enhance the anti‐AD efficacy of tolerogenic adjuvants through rational optimization of the adjuvant composition and the treatment regimen, including route and frequency of administration.

